# Supplementary figures and images for: Numerical dispersed flow simulation of fire-flake particle dynamics and its learning representation
Source: PeerJ Comput Sci. 2025 Apr 22;11:e2836. doi: 10.7717/peerj-cs.2836 (PMC12190462; doi:10.7717/peerj-cs.2836)

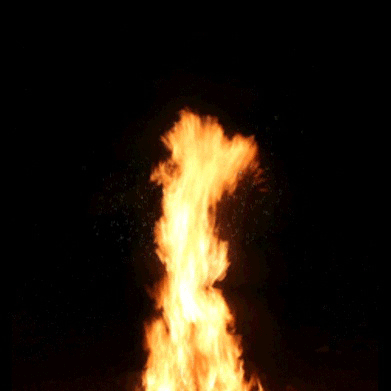

Supplement: Supplemental Information 2 — A scene where we experimented with reading a *.gif file and generating a fire-flake from it. [file peerj-cs-11-2836-s002.zip › result1.gif]

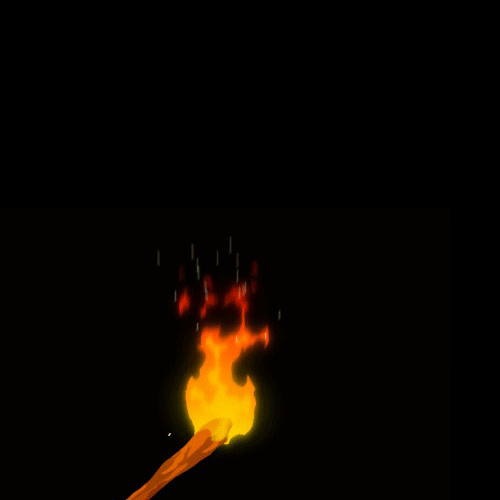

Supplement: Supplemental Information 2 — A scene where we experimented with reading a *.gif file and generating a fire-flake from it. [file peerj-cs-11-2836-s002.zip › result3.gif]
